# Effects of moisture content on the co-oxidation behavior of sturgeon myofibrillar protein and sturgeon oil

**DOI:** 10.1016/j.fochx.2026.104481

**Published:** 2026-09-16

**Authors:** Yizhou Fang, Zhengmei Yin, Jinshui Yu, Guangrong Huang, Junhui Li

**Affiliations:** a Key Laboratory of Marine Food Quality and Hazard Controlling Technology of Zhejiang Province, College of Life Sciences, China Jiliang University, Hangzhou, Zhejiang 310018, PR China; b School of Food Science and Engineering, Hangzhou Medical College, Hangzhou, Zhejiang 310059, PR China

**Keywords:** Myofibrillar protein, Sturgeon oil, Moisture content, Protein–lipid co-oxidation, Reactive oxygen species

## Abstract

This study systematically investigated the moisture-dependent regulation of marine protein–lipid co-oxidation across a broad moisture range (10–90%), with particular emphasis on the poorly understood intermediate-moisture region. Protein and lipid oxidation exhibited a non-linear response to increasing moisture content, first intensifying and then declining, with the most extensive co-oxidation observed at 70% MC. Moderate free water promotes co-oxidation by enhancing molecular mobility and reactive species generation, while low (≤30%) or high (≥90%) moisture suppresses it via restricted mobility or dilution/oxygen limitations, respectively. These findings suggest a moisture-dependent transition in protein–lipid co-oxidation and the potential existence of a critical threshold, most likely within the 40–60% moisture range. Moreover, validation in real sturgeon matrices demonstrated excellent agreement between the temporal changes observed in the model and those in the real samples, supporting the applicability of the model-derived conclusions to actual products and providing a basis for improving the oxidative stability of marine protein products.

## 1 Introduction

Marine protein products, including surimi, soluble fish protein powders, and various restructured marine protein foods, play an important role in the global food supply chain owing to their high protein content and distinctive nutritional properties. However, these products are highly susceptible to quality deterioration during processing, storage, and distribution, mainly manifested as declines in gel properties, water-holding capacity, and sensory quality (Shahidi & Saeid, 2025; Yan et al., 2024). Although frozen storage can substantially improve the storage stability of marine protein products, the formation of ice crystal during freezing may cause mechanical damage to the protein network and induce protein denaturation and aggregation, thereby impairing the quality of protein products. In addition, repeated freeze–thaw cycles may further promote the oxidation of protein and lipid, leading to the structural deterioration of protein (Jin et al., 2025; Wang, Chen, et al., 2025). Therefore, under practical cold-chain distribution and short-term low-temperature storage conditions, the stability of marine protein products during refrigerated storage at 4 °C remains a critical factor affecting quality preservation.

Numerous studies have shown that myofibrillar proteins are the major functional protein components in protein-based products, and their structural integrity and functional stability directly determine key quality attributes such as gel properties, emulsifying capacity, and water-holding capacity (Jiang et al., 2021; Zhang et al., 2023). Meanwhile, proteins and lipids commonly coexist in marine protein products, and their co-oxidation behavior is a critical factor governing product quality stability (Chen et al., 2022; Li et al., 2024). In protein products, protein oxidation and lipid oxidation promote each other and co-evolve synergistically through free radical chain reactions and cross-linking induced by secondary oxidation products. Specifically, lipid hydroperoxides and their degradation products, such as malondialdehyde and 4-hydroxynonenal, can react with amino acid residues in protein side chains, inducing protein carbonylation, cross-linking, aggregation, and reduced solubility. Conversely, radicals generated during protein oxidation can also initiate lipid peroxidation chain reactions (Chen et al., 2023; Liao et al., 2026). This co-oxidation process is particularly pronounced in marine protein products, as marine lipids are rich in polyunsaturated fatty acids and are therefore more susceptible to oxidation than many terrestrial lipids, thereby markedly aggravating oxidative damage to proteins (Fang et al., 2020). Therefore, elucidating the molecular mechanisms underlying the co-oxidation of marine myofibrillar proteins and lipids is of particular scientific significance for improving the oxidative and storage stability of marine protein products.

Water, as the most abundant component in marine protein products, plays a dual and complex role in protein–lipid co-oxidation. In low-moisture systems, the effects of water plasticization and molecular mobility on oxidation rates have been preliminarily recognized; whereas in high-moisture emulsions, the aqueous phase serves as a diffusion medium for pro-oxidant metal ions and free radicals, thereby markedly affecting interfacial oxidation kinetics (Ghelichi et al., 2023; Wang, Wu, et al., 2025). Meissner et al. (2020) also reported that, in whey protein-based oleogels, increasing water addition from 0.23% to above 2.4% caused a significant increase in both lipid hydroperoxide formation and protein oxidation degree. Studies on protein–lipid co-oxidation under different moisture conditions have mainly focused on two types of systems: low-moisture powdered systems (Fang et al., 2023), typically with moisture contents below 30%, and high-moisture solution or emulsion systems (Chen et al., 2023; Hu et al., 2025), generally with moisture contents above 90%. More recently, increasing attention has been directed toward protein and lipid oxidation in intermediate-moisture foods (Dietrich et al., 2025; Guo et al., 2021), reflecting the practical importance of products such as semi-moist restructured fish products, ready-to-eat marine protein snacks, and certain prepared protein gel products. However, most of these studies have focused on a single intermediate-moisture formulation or on relatively narrow moisture variations within a specific product matrix, typically spanning no more than approximately 30 percentage points (e.g., 50–80% moisture content) (Guo et al., 2021). However, systematic investigations covering a broad moisture range within a unified protein–lipid system remain scarce. A broader moisture gradient is needed to identify non-linear oxidation behavior and potential transition regions that may be overlooked when only a narrow moisture range is examined. In particular, it is still unclear how large shifts in moisture content alter water state, molecular mobility, reactive oxygen species generation, and the dynamic coupling between lipid oxidation and protein oxidation across low-, intermediate-, and high-moisture regimes. Therefore, systematically elucidating the effects of moisture content across a broad range on marine protein–lipid co-oxidation is of considerable theoretical and practical significance for identifying moisture-dependent oxidation patterns and guiding the development, formulation, processing, and storage of marine protein products.

Besides, sturgeon is an economically important aquaculture species worldwide, with its roe being processed into caviar, one of the most highly valued aquatic delicacies. In addition to roe, sturgeon meat, accounting for approximately 40–50% of body weight, constitutes a substantial edible resource and is commercially processed into various products, including frozen fillets, smoked products, sausages, and surimi, with annual global production exceeding 100,000 t (Fang et al., 2026). However, sturgeon meat is rich in unsaturated fatty acids, with polyunsaturated fatty acids (PUFAs) accounting for approximately 40% of the total fatty acids (Raposo et al., 2023), rendering it particularly susceptible to oxidative deterioration during processing and storage.

Therefore, this study employed sturgeon myofibrillar protein and sturgeon oil as representative marine protein and lipid sources to establish a ternary “protein–lipid–water” co-oxidation model with varying moisture contents. Rather than simply characterizing oxidation at different moisture levels, we sought to determine whether moisture regulates protein–lipid co-oxidation through distinct mechanisms across different moisture ranges. By integrating changes in protein and lipid oxidation with water activity and distribution, free radical generation, and protein structural alterations, we aimed to unravel the moisture-dependent interplay among water status, radical reactions, and protein–lipid oxidation, and to identify the key factors governing co-oxidation at different moisture levels. These findings are expected to deepen the mechanistic understanding of how water modulates protein–lipid co-oxidation and provide a theoretical basis for targeted moisture regulation to improve the oxidative stability of marine protein products.

## 2 Materials and methods

### 2.1 Raw materials

Hybrid sturgeon (*Acipenser schrenckii* as the maternal line and *Acipenser baerii* as the paternal line; 4–5 years old, approximately 1.3 m in body length and 9 kg in body weight) were purchased from Sishui Haiquan Aquatic Products Co., Ltd. This hybrid was selected because it is a commercially important and widely cultured sturgeon in China, with favorable growth performance and a muscle lipid profile rich in unsaturated fatty acids (Yang et al., 2025), making it a representative material for investigating protein–lipid oxidative interactions. The fish were transported and stored at −18 °C until use. Other chemical reagents and assay kits were purchased from Macklin Biochemical Co., Ltd. (Shanghai, China) and Yuanye Bio-Technology Co., Ltd. (Shanghai, China).

### 2.2 Preparation of the protein–lipid–water ternary system

#### 2.2.1 Sturgeon myofibrillar protein

Sturgeon myofibrillar protein was prepared according to the method of Wang et al. (2025a) with slight modifications. Briefly, the dorsal muscle of sturgeon was dissected, collected, and minced. Four volumes of low-salt phosphate buffer (20 mmol/L phosphate-buffered saline, 50 mmol/L NaCl, pH 7.0) were added to the minced muscle and thoroughly mixed. The homogenate was centrifuged at 8000 rpm for 15 min at 4 °C, and the supernatant was discarded. This washing procedure was repeated once. Subsequently, four volumes of low-salt phosphate buffer were added to the precipitate, followed by homogenization and filtration to remove connective tissue. The filtrate was mixed with four volumes of cold distilled water and centrifuged to collect the precipitate. This procedure was repeated three times, and the resulting precipitate was collected as sturgeon myofibrillar protein.

#### 2.2.2 Sturgeon oil

Sturgeon oil was extracted by Soxhlet extraction according to the method of Fang et al. (2023) with slight modifications. Briefly, sturgeon liver was minced and extracted with n-hexane at 90 °C for 24 h. The n-hexane was subsequently removed using a rotary evaporator at 40 °C and 100 rpm.

#### 2.2.3 Ternary system

To investigate the effect of moisture content on the co-oxidation process of sturgeon myofibrillar protein and lipid, ternary systems with different moisture contents were prepared and stored at 4 °C. Sturgeon oil was added to sturgeon myofibrillar protein to prepare a binary protein–lipid system containing 10% lipid. The mixture was then evenly divided into five portions, and different amounts of water were added to obtain protein–lipid–water ternary systems with moisture contents of 10%, 30%, 50%, 70%, and 90%, which were designated as 10% MC, 30% MC, 50% MC, 70% MC, and 90% MC, respectively.

#### 2.2.4 Real sturgeon meat samples

To evaluate whether the findings obtained from the ternary model system could be extrapolated to a complex food matrix, two additional sturgeon muscle-based samples were prepared. The first sample, designated as native sturgeon meat (NSM), consisted of fresh sturgeon meat that was subjected only to mincing without any further processing, thereby representing the natural food matrix. However, as shown in Table s1 of supplementary materials, the protein-to-lipid ratio of fresh sturgeon meat differed substantially from that of the protein–lipid–water ternary systems investigated in this study. Therefore, a second sturgeon meat-based sample, designated as composition-matched sturgeon meat (CSM), was prepared by supplementing the minced sturgeon meat with isolated sturgeon myofibrillar protein and deionized water. As summarized in Table s1, the moisture, protein, and lipid contents of the CSM did not differ significantly (*p* > 0.05) from those of the 70% MC. Subsequently, both the NSM and CSM samples were subjected to the same γ-irradiation and oxidative storage conditions as those applied to the 70% MC model system.

#### 2.2.5 γ-irradiation sterilization

To eliminate the potential interference of microbial activity in the co-oxidation process and to specifically investigate the physicochemical regulatory effects of moisture, all samples were subjected to γ-irradiation sterilization (Wei et al., 2022). Irradiation was performed using a BFT irradiation facility (Nordion, Canada) with Co-60 as the radiation source at a dose of 10 kGy. It should be noted that γ-irradiation may induce certain physicochemical changes in proteins and lipids. However, both the prepared protein and lipid were subjected to the same irradiation treatment before being formulated into ternary model systems with different moisture contents; therefore, the potential initial effects caused by irradiation were considered consistent among treatments. In addition, previous studies have reported that an absorbed dose of 10 kGy has only a limited effect in protein oxidation and structural characteristics (Stanca et al., 2023), which is consistent with the results obtained in the present study, as shown in Table S2 of the supplementary materials. After sterilization, the samples were immediately transferred to a sterile workstation. All instruments and packaging materials in contact with the samples were sterilized by autoclaving at 121 °C for 20 min to maintain aseptic conditions throughout the oxidation experiment.

Under sterile conditions, protein–lipid–water samples with different moisture contents were packed into sealed bags (15 g per bag) or 10 mL centrifuge tubes and stored at 4 °C for oxidation. Samples were collected every 7 d for analysis. Three packaged samples were randomly selected for chemical composition analysis, and the results are shown in Table 1. The measured contents of protein, lipid, and moisture were consistent with the expected values, also indicating that the mixture was homogeneous. In addition, because the actual protein and lipid contents differed among samples with different moisture contents, the sampling amount for each assay was adjusted according to moisture content to ensure that the absolute mass of protein or lipid was consistent among samples.

**Table 1:** Protein and lipid oxidation indices under different moisture contents.

Sample	Protein oxidation index	Lipid oxidation index	Protein/Lipid
10%MC	0.228 ± 0.004^f^	6.181 ± 0.074^f^	0.037 ± 0.001^f^
30%MC	0.408 ± 0.017^e^	6.991 ± 0.226^e^	0.058 ± 0.004^e^
50%MC	0.852 ± 0.025^c^	7.587 ± 0.072^d^	0.112 ± 0.003^b^
70%MC	1.035 ± 0.026^b^	8.168 ± 0.324^c^	0.127 ± 0.008^a^
90%MC	0.524 ± 0.018^d^	4.968 ± 0.080^g^	0.106 ± 0.005^c^
Native sturgeon meat	1.756 ± 0.093^a^	21.242 ± 0.727^a^	0.083 ± 0.006^d^
Composition-matched sturgeon meat	1.666 ± 0.057^a^	16.144 ± 0.861^b^	0.103 ± 0.005^c^

Different letters indicate significant differences among groups (*p* < 0.05).

### 2.3 Assessment of protein and lipid oxidation

#### 2.3.1 Protein oxidation

##### 2.3.1.1 Sulfhydryl content

Total sulfhydryl (T-SH) and reactive sulfhydryl (R-SH) contents were determined according to the method of Fang et al. (2023). For T-SH determination, samples containing 100 mg of protein were homogenized in 10 mL of Tris-HCl buffer containing 0.2 mol/L Tris-HCl, 2% SDS, 10 mmol/L EDTA, and 8 mol/L urea at pH 8.0. After thorough mixing, the homogenate was centrifuged at 10,000 rpm for 10 min. Subsequently, 3 mL of supernatant was mixed with 50 μL of Ellman's reagent (10 mmol/L DTNB), incubated at room temperature in the dark for 10 min, and the absorbance was measured at 412 nm. For R-SH determination, the same procedure was used except that urea was omitted from the Tris-HCl buffer. The sulfhydryl content was calculated using the following equation:

$$T-\mathrm{SH}\;\text{or}\;R-\mathrm{SH}\;\left(\text{nmol}/\mathrm{mg}\right)=A₄₁₂\times 10^{6}/\left(1.36\times 10^{4}\right)$$

##### 2.3.1.2 Carbonyl content

The carbonyl content of the samples was determined using a commercial P930765 assay kit (Macklin, Shanghai, China).

#### 2.3.2 Lipid oxidation

##### 2.3.2.1 Acid value and peroxide value

The acid value (AV) and peroxide value (POV) of the samples were determined according to AOCS official methods Cd 3d-63 and Cd 8b-90, respectively (AOCS, 2009). For AV determination, 5 g of extracted oil was dissolved in 100 mL of neutralized toluene–isopropanol (1:1, *v*/v) and titrated with 0.1 mol/L KOH using phenolphthalein as the indicator until a faint pink color persisted for 30 s. For POV determination, 5 g of oil was dissolved in 30 mL of acetic acid–isooctane (3:2, *v*/v), mixed with 0.5 mL of saturated KI, and reacted for 1 min. After adding 30 mL of distilled water, the liberated iodine was titrated with standardized sodium thiosulfate (0.1 mol/L) using starch as the endpoint indicator.

##### 2.3.2.2 Malondialdehyde content

The malondialdehyde (MDA) content, representing secondary lipid oxidation products, was determined using an R21870 MDA assay kit (Yuanye, Shanghai, China).

#### 2.3.3 Protein oxidation index and lipid oxidation index

To comprehensively evaluate the overall changes in protein oxidation and lipid oxidation under different moisture contents, the area under the curve (AUC) was calculated based on the relative change rates of oxidation indicators over time, according to the method of Bakir et al. (2017) with modifications. Protein oxidation index (POI) was defined as the mean AUC of T-SH loss rate, R-SH loss rate, and carbonyl increase rate. Lipid oxidation index (LOI) was defined as the mean AUC of AV, POV, and MDA increase rates.

### 2.4 Moisture characteristics

#### 2.4.1 Water activity

Water activity was measured using a AquaLab Series 4TE water activity meter (METER Group, USA) according to the method described by Campbell et al. (2022), with minor modifications. 5 g of each sample was placed evenly into the sample cup under 25 °C and equilibrated in the sealed chamber until a stable reading was obtained. Measurements were performed in triplicate.

#### 2.4.2 Water distribution

Water distribution was determined using a nuclear magnetic resonance (NMR) imaging analyzer (MacroMR12–60, Niumag Corporation, China) according to Jiang et al. (2025). After equilibrating 100 mg of sample to room temperature, the sample was transferred into an NMR tube. The NMR parameters were set as follows: resonance frequency, 12 MHz; 90° radiofrequency pulse width, 7.4 μs; 180° radiofrequency pulse width, 11.6 μs; number of echoes, 20,000; and echo time, 0.9 ms. The transverse relaxation time data were processed using the Sirt inversion algorithm to analyze water composition in the samples.

### 2.5 Protein structural

#### 2.5.1 Secondary structure

Protein secondary structure was determined using a portable Raman spectrometer (QE-Pro, Ocean Optics, USA) according to Peters et al. (2025). The samples were evenly spread on the detection platform over an area of approximately 1 cm^2^. The excitation wavelength was set at 785 nm, laser power at 300 mW, integration time at 5 s, and scanning range at 200–2000 cm^−1^. Each sample was scanned three times. The raw spectra were processed by baseline correction, smoothing, and normalization. The amide I band region (1600–1700 cm^−1^) was fitted to calculate the relative contents of α-helix, β-sheet, β-turn, and random coil structures.

#### 2.5.2 Tertiary structure

Intrinsic fluorescence spectra were measured using a fluorescence spectrophotometer (F-4600, Hitachi, Japan) according to the method of Teng et al. (2022) with slight modifications. Samples containing 100 mg of protein were diluted to 6 mL with 10 mmol/L phosphate buffer. The excitation wavelength was set at 283 nm, and emission spectra were recorded from 300 to 430 nm. The excitation and emission slit widths were both set at 5 nm. Changes in the maximum emission wavelength and fluorescence intensity were used to evaluate protein tertiary structure.

### 2.6 Free radical levels

Superoxide anion radicals (O₂•^−^) and hydroxyl radicals (•OH) generated during oxidation were detected using electron paramagnetic resonance (EPR) spectroscopy (EPR200M, CIQTEK, China) combined with spin-trapping technology (Misak et al., 2018). For O₂•^−^ detection, to avoid competitive inhibition of spin-trapping efficiency by the aqueous phase, 50 mg of freeze-dried sample was mixed with 1 mL of methanol–phosphate buffer (9:1, *v*/v) containing 50 mmol/L 5-Ethoxycarbonyl-5-methyl-1-pyrroline N-oxide as the spin-trapping agent. The mixture was vortexed and incubated in the dark at 25 °C for 15 min. The reaction solution was filtered through a 0.22 μm organic membrane and transferred into a quartz capillary tube for EPR analysis. For •OH detection, the reaction was conducted in an aqueous system. Briefly, 50 mg of freeze-dried sample was mixed with 1 mL of phosphate buffer (50 mmol/L, pH 7.4) containing 100 mmol/L 5,5-Dimethyl-1-pyrroline N-oxide as the spin-trapping agent. The mixture was vortexed and incubated in the dark at 25 °C for 10 min, filtered through a 0.22 μm membrane, and transferred into a quartz capillary tube for EPR analysis. The EPR parameters were set as follows: center magnetic field, 3350 G; sweep width, 100 G; microwave frequency, 9.86 GHz; microwave power, 10 mW; modulation frequency, 100 kHz; modulation amplitude, 1.0 G; time constant, 40 ms; scan time, 60 s; and number of accumulations, 3.

### 2.7 Statistical analysis

All experimental results are expressed as mean ± standard error. Each group was analyzed in triplicate. Statistical analysis was performed using SPSS software (version 23.0, USA). One-way analysis of variance was used to evaluate differences among groups, and *p* < 0.05 was considered statistically significant. Pearson correlation coefficients were calculated to assess relationships among different indicators. Principal component analysis (PCA) was further conducted using SPSS (version 23.0, USA) on standardized data to characterize the multivariate relationships among the measured indicators. Principal components were extracted from the correlation matrix, and those with eigenvalues greater than 1.0 were retained for subsequent analysis.

## 3 Results and discussion

### 3.1 Macroscopic morphology

The morphological changes in samples with different moisture contents after 35 d of oxidation at 4 °C are shown in Fig. 1. At the initial time point (0 d), the physical states of the samples differed markedly depending on moisture content: low-moisture samples appeared as powders, intermediate-moisture samples exhibited a paste-like state, whereas high-moisture samples were completely transformed into solution-like systems. This phenomenon may be attributed to the progressive penetration of water molecules into the intermolecular spaces of proteins with increasing moisture content, which promoted protein chain expansion and pronounced swelling (Meissner et al., 2020), ultimately resulting in powdery, paste-like, or colloidal dispersion systems. In addition, water exudation was observed to varying degrees in all samples as storage time increased. For the powdery and paste-like samples, apparent viscosity increased markedly; in particular, the 50% MC sample changed from an initial powdery state to a nearly paste-like form. Water exudation may be associated with excessive protein oxidation, cross-linking, and degradation, which weaken intermolecular interactions among proteins, induce collapse of the spatial structure, and consequently release water originally retained within the protein matrix (Maidannyk et al., 2022).

**Fig. 1:**
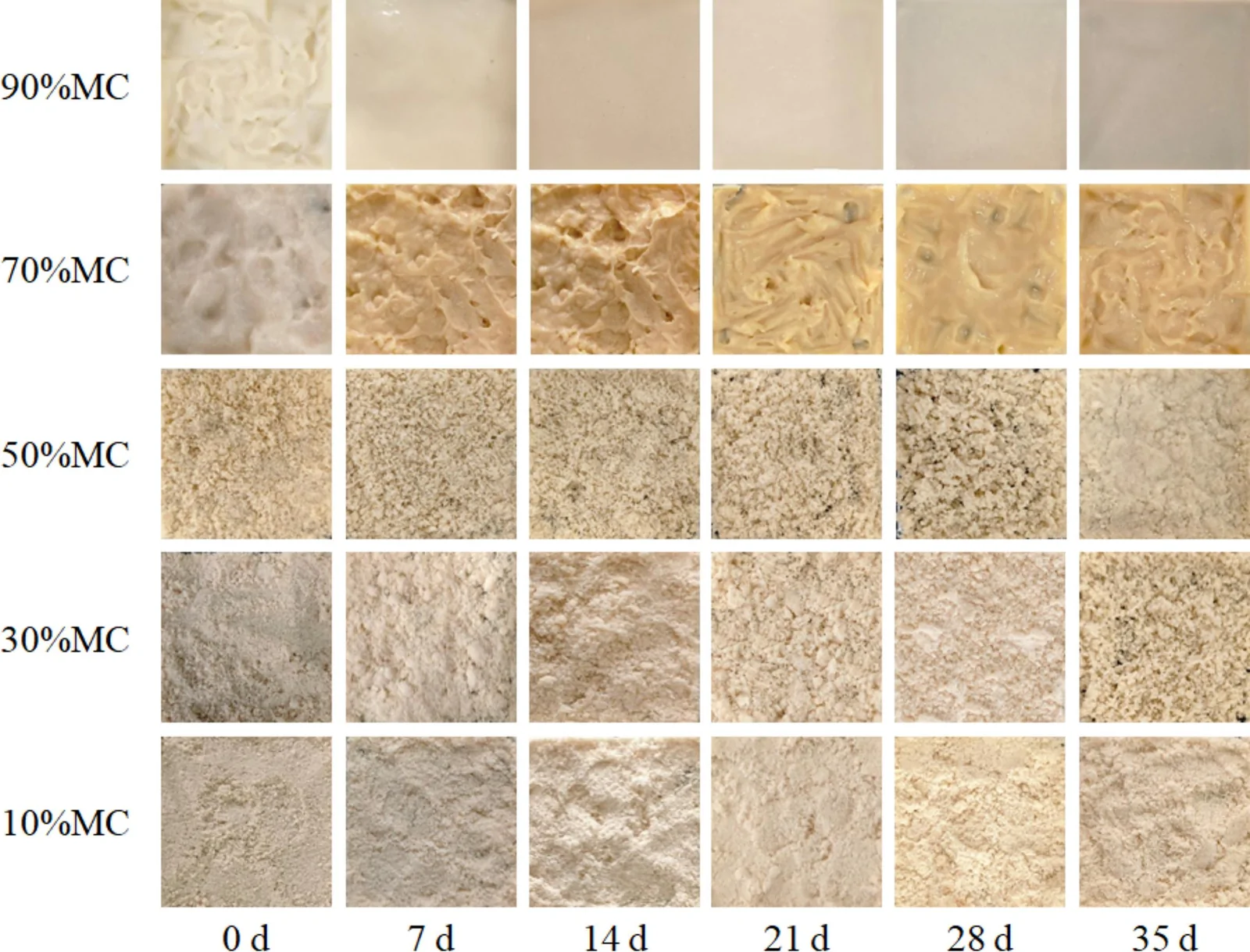
Moisture-dependent changes in the visual appearance of the protein–lipid-water model systems during 35 d of storage at 4 °C.

### 3.2 Oxidation degree

To investigate the effects of moisture content on protein–lipid co-oxidation, protein and lipid oxidation levels in the samples were analyzed during storage. Protein oxidation was characterized by T-SH, R-SH, and carbonyl contents, whereas lipid oxidation was evaluated by AV, POV, and MDA content. The corresponding results are shown in Fig. 2 A–F, and the relative change rates of each indicator are provided in Fig. S1 A–F in the supplementary materials.

**Fig. 2:**
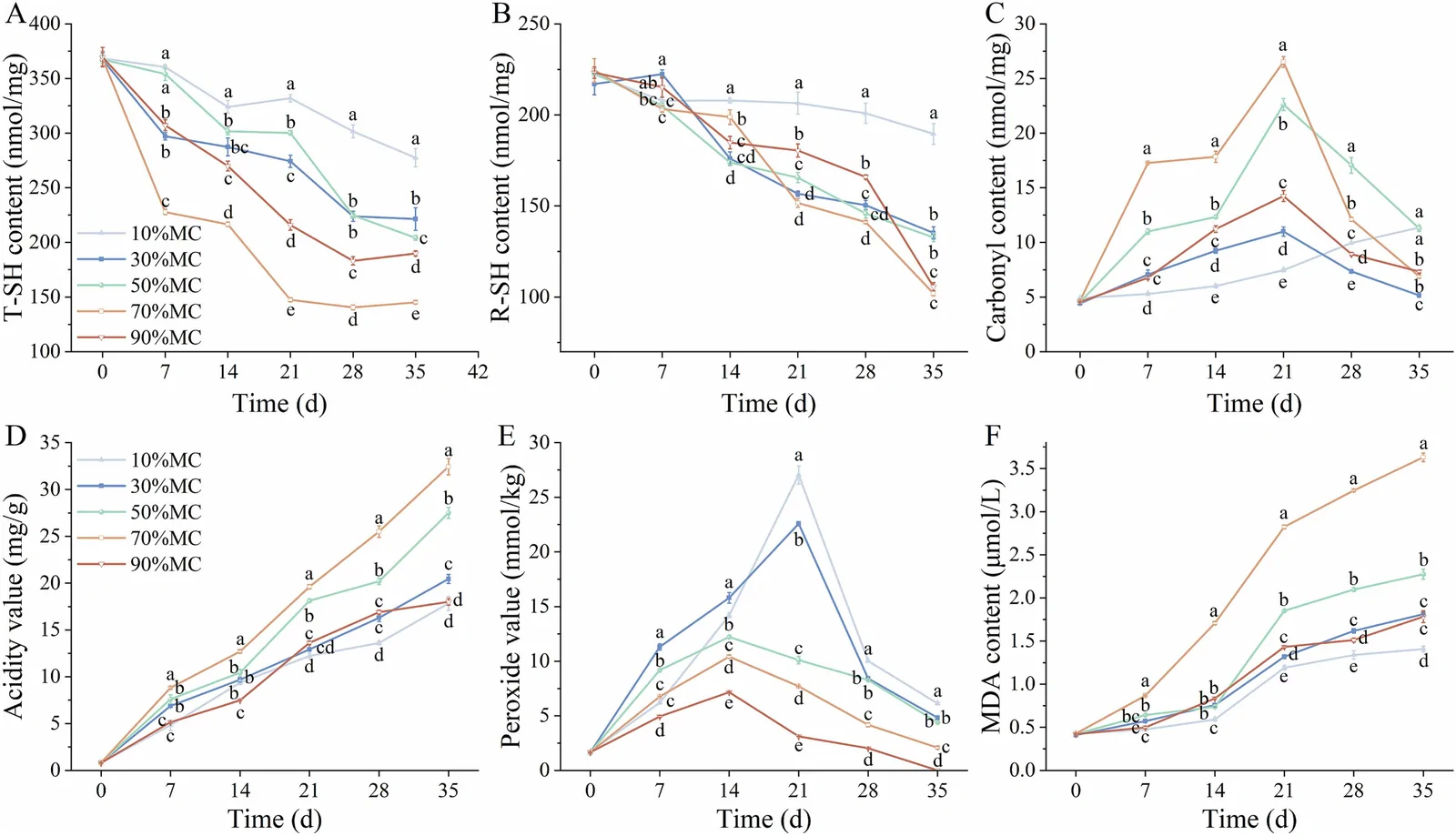
Effects of moisture content on the time-dependent changes in protein and lipid oxidation during storage. (A) Total sulfhydryl content; (B) reactive sulfhydryl content; (C) carbonyl content; (D) acid value; (E) peroxide value; and (F) malondialdehyde content. Different letters indicate significant differences among groups (*p* < 0.05).

#### 3.2.1 Oxidation degree of protein

##### 3.2.1.1 Sulfhydryl content

With increasing oxidation time, both T-SH and R-SH contents decreased continuously in all samples, which is consistent with previous studies on protein oxidation (Cui et al., 2019; Fang et al., 2023). Oxidation gradually induced unfolding of the protein molecular structure, allowing free radicals to attack exposed sulfhydryl groups and form oxidation products such as disulfide bonds, ultimately reducing the measurable sulfhydryl content (Dominguez et al., 2022). Notably, the R-SH content of the 30% MC sample slightly increased at 7 d before decreasing continuously. This may be attributed to mild oxidation during the early oxidation stage, which induced partial protein unfolding and exposed sulfhydryl groups originally buried inside the protein structure, temporarily increasing the detectable R-SH content (Fang et al., 2023).

In addition, the sulfhydryl decay rates of the 70% and 90% MC were significantly higher than those of the other samples (*p* < 0.05). The decreasing proportions in the 50% MC and 30% MC were comparable, whereas the 10% MC showed the lowest sulfhydryl loss. Specifically, in 70% MC and 90% MC, the decay rate of T-SH was 61% and 49%, respectively, whereas that of R-SH was approximately 50% in both cases. In contrast, T-SH and R-SH in the 10% MC sample decreased by only 25% and 15%, respectively. Previous studies have suggested that, under high-moisture conditions, water molecules can disrupt the compact protein structure by competing for hydrogen bonds and weakening hydrophobic interactions (Lavelli & Sereikaite, 2022; Zhou et al., 2025), thereby promoting protein unfolding and sulfhydryl exposure. Meanwhile, water can act as a medium for free radical generation and migration (Ruzza et al., 2021), accelerating radical attack on exposed sulfhydryl groups and leading to a more pronounced decrease in sulfhydryl content. However, the sulfhydryl decay rate of the 90% MC was significantly lower than that of the 70% MC (*p* < 0.05), which may be because excessive water not only diluted oxygen, free radicals, and other oxidative intermediates, but also restricted oxygen diffusion toward the protein interface (Tran et al., 2020), thereby attenuating sulfhydryl oxidative loss.

##### 3.2.1.2 Carbonyl content

As shown in Fig. 2 C, except for the 10% MC, the carbonyl content of all samples increased with oxidation time before 21 d and then began to decline. Carbonyl accumulation results from peptide bond cleavage and direct modification of amino acid side chains during protein oxidation, whereas the subsequent decrease may be associated with further oxidation of carbonyl groups, volatilization, and physical masking by protein aggregates (Choudhury et al., 2025; Nawaz et al., 2022). In contrast, the carbonyl content of the 10% MC increased continuously, possibly because the relatively low oxidation degree limited carbonyl consumption, allowing the formation rate to remain higher than the consumption rate.

The peak carbonyl contents under intermediate- and high-moisture conditions followed the order 70% MC > 50% MC > 90% MC > 30% MC. Notably, although the 90% MC had the highest moisture content, its side-chain oxidation degree was lower than those of the 70% and 50% MC, which was likely associated with the dilution effect of excessive water and restricted oxygen diffusion, as discussed in Section 3.2.1.1. These results further suggest that, in the high-moisture range, the inhibitory effects of dilution and oxygen diffusion limitation on side-chain oxidation may even outweigh the promoting effect of water activity itself. In addition, the decline in carbonyl content after 21 d was markedly slower in the 50% MC than in the 70% MC, resulting in a higher carbonyl content at 35 d. This may be attributed to the high viscosity of the paste-like system (Dominguez et al., 2022), which limited the diffusion of free radicals and oxygen, thereby slowing carbonyl consumption.

#### 3.2.2 Oxidation degree of lipid

##### 3.2.2.1 Av

AV is an important indicator of the amount of free fatty acids generated in lipids. The initial AV of the oil was approximately 0.8 mg/g, meeting the requirement for fresh fish oil (Fang et al., 2018). In addition, AV increased with oxidation time in all samples. This may be attributed to the hydrolysis of triglycerides into free fatty acids and the oxidative degradation of unsaturated fatty acids, during which intermediates such as hydroperoxides are formed and subsequently decomposed into free fatty acids, consistent with previous findings (Fang et al., 2023; Xu et al., 2021). In addition, residual endogenous enzymatic activity may also have contributed to AV development, as a previous study reported that some hydrolytic enzymes, including lipases, can retain appreciable activity after γ-irradiation at approximately 10 kGy (Kim et al., 2007).

AV was generally higher in the 70% and 50% MC samples than in the other moisture groups, with the 70% MC showing the greatest increase. Previous studies have suggested that changes in AV may be associated with the influence of water activity on enzymatic hydrolysis, as enzymes require an appropriate amount of free water to maintain their active conformation (Erickson et al., 2023). In addition, the paste-like state of the 50% MC may have limited the contact efficiency between enzymes and substrates, thereby restricting the increase in AV. Notably, although the moisture contents of the 10% MC, 30% MC, and 90% MC differed markedly, their AV increase rates remained similar. For the 10% MC and 30% MC samples, low water activity may have limited the interfacial activation of lipase, while the powdery state further restricted the diffusion and contact between enzymes and substrates (Dietrich et al., 2025), thereby suppressing enzymatic hydrolysis. By contrast, although the water activity of the 90% MC was similar to that of the 70% MC and 50% MC, excessive water may have formed an overly thick hydration layer around enzyme molecules (Bülbül et al., 2020), leading to conformational instability or reduced interfacial accessibility, ultimately limiting hydrolysis efficiency.

#### 3.2.3 POV and MDA

POV and MDA content reflect the accumulation of primary and secondary lipid oxidation products, respectively. As shown in Fig. 2 E and F, POV exhibited an initial increase followed by a subsequent decrease under all moisture conditions, whereas MDA content increased continuously with oxidation time, consistent with previous study (Jaldani et al., 2024). During the early stage of oxidation, free radical chain reactions promoted the rapid accumulation of primary oxidation products, resulting in an increase in POV. As oxidation progressed, these primary oxidation products were further decomposed into secondary oxidation products, leading to a decline in POV (Pashaei & Farhoosh, 2025). For POV, the 30% MC showed the highest increase rate during the early oxidation stage, followed by the 50% and 70% MC; these three groups reached their peak values after 14 or 21 d and then began to decline. In contrast, MDA accumulated most rapidly in the 70% MC sample throughout storage, whereas the other moisture groups showed comparatively slower increases. Previous study has shown that free radical chain reactions can induce an exponential increase in the formation rate of secondary lipid oxidation products (Erickson et al., 2023), which is highly consistent with the observed trend and coincides with the onset of POV decline in the high-moisture samples.

By integrating the changes in POV and MDA content, it can be inferred that the dominant lipid oxidation pathways differed markedly among moisture conditions. These results suggest that secondary lipid oxidation was particularly pronounced at 70% MC, where primary oxidation products appeared to be rapidly converted into secondary oxidation products. In contrast, lipid oxidation in the 10% MC was dominated by primary oxidation, as indicated by its highest POV peak but the slowest MDA accumulation. These results are consistent with previous findings showing that water can influence oxidation pathways through interfacial effects and plasticization, with higher water activity generally favoring the formation of secondary oxidation products (Amakhmakh et al., 2024). In addition, the 90% MC, which had the highest moisture content, exhibited relatively low-to-intermediate levels of both POV and MDA. This may be because excessive water filled internal voids and formed an aqueous layer within the system, thereby restricting oxygen and free radical diffusion (Bülbül et al., 2020) and ultimately reducing lipid oxidation under high-moisture conditions.

#### 3.2.4 Co-oxidation process of protein and lipid

Considering that some oxidation indicators exhibited dynamic changes characterized by an initial increase followed by a subsequent decrease during the oxidation process, values measured at a single time point may not accurately reflect the overall oxidation process. Therefore, based on the AUC of standardized relative change rates (Bakir et al., 2017), the POI and LOI were further established to comprehensively characterize the dynamic changes in protein–lipid co-oxidation under different moisture contents, which are shown in Table 1.

First, LOI was consistently much higher than POI throughout the oxidation process, indicating that lipids exhibited greater sensitivity to oxidation and stronger cumulative oxidative changes than proteins. This result is consistent with previous study and may be attributed to the higher susceptibility of lipids, especially unsaturated fatty acids, to attack by oxygen and free radicals (Fang et al., 2023). In addition, as moisture content increased from 10% MC to 70% MC, both POI and LOI gradually increased and reached their maximum values at 70% MC, with values of 1.035 and 8.168, respectively. However, when moisture content further increased to 90% MC, both integrated oxidation indices decreased. As discussed in Sections 3.2.1 and 3.2.2, an appropriate amount of water markedly promoted protein and lipid oxidation, whereas excessively low or high moisture contents were unfavorable for the sustained progression of oxidation reactions.

On the other hand, protein oxidation may, to some extent, be induced and regulated by lipid oxidation products (Zhang et al., 2024). Therefore, the POI/LOI ratio can be used to characterize the response of protein oxidation relative to lipid oxidation, namely the potential influence of lipid oxidation on protein oxidation. The POI/LOI ratio was also highest at 70% MC, indicating a stronger relative response of protein oxidation to lipid oxidation under this moisture condition. This may be because an appropriate amount of water enhanced molecular mobility, free radical diffusion, and interactions between proteins and lipid oxidation products within the system (Zhang et al., 2025), thereby promoting protein–lipid co-oxidation. Notably, the POI/LOI ratio of the 90% MC was close to those of the 50% MC and 70% MC, suggesting that when moisture content exceeded 50%, the inducing effect of lipid oxidation on protein oxidation tended to stabilize. However, the absolute levels of POI and LOI in the 90% MC were relatively low, indicating that excessive water exerted a more pronounced inhibitory effect on lipid oxidation, thereby limiting lipid oxidation product-mediated protein oxidation.

To further elucidate the causes of the above oxidation differences, subsequent analyses were conducted from three aspects: water activity and water distribution, protein structural changes, and free radical generation, providing mechanistic verification and interpretation.

### 3.3 Moisture characteristics

#### 3.3.1 Water activity

The changes in moisture content and water activity of different samples were determined, as shown in Fig. s2 of supplementary materials and Fig. 3 A, respectively. As shown in Fig. s2, the moisture contents of all samples met the requirements of the experimental design and showed only slight, non-significant fluctuations during oxidation (*p* > 0.05). This was mainly due to the low experimental temperature, under which water evaporation was extremely slow, allowing the moisture content to remain relatively stable throughout the oxidation process.

**Fig. 3:**
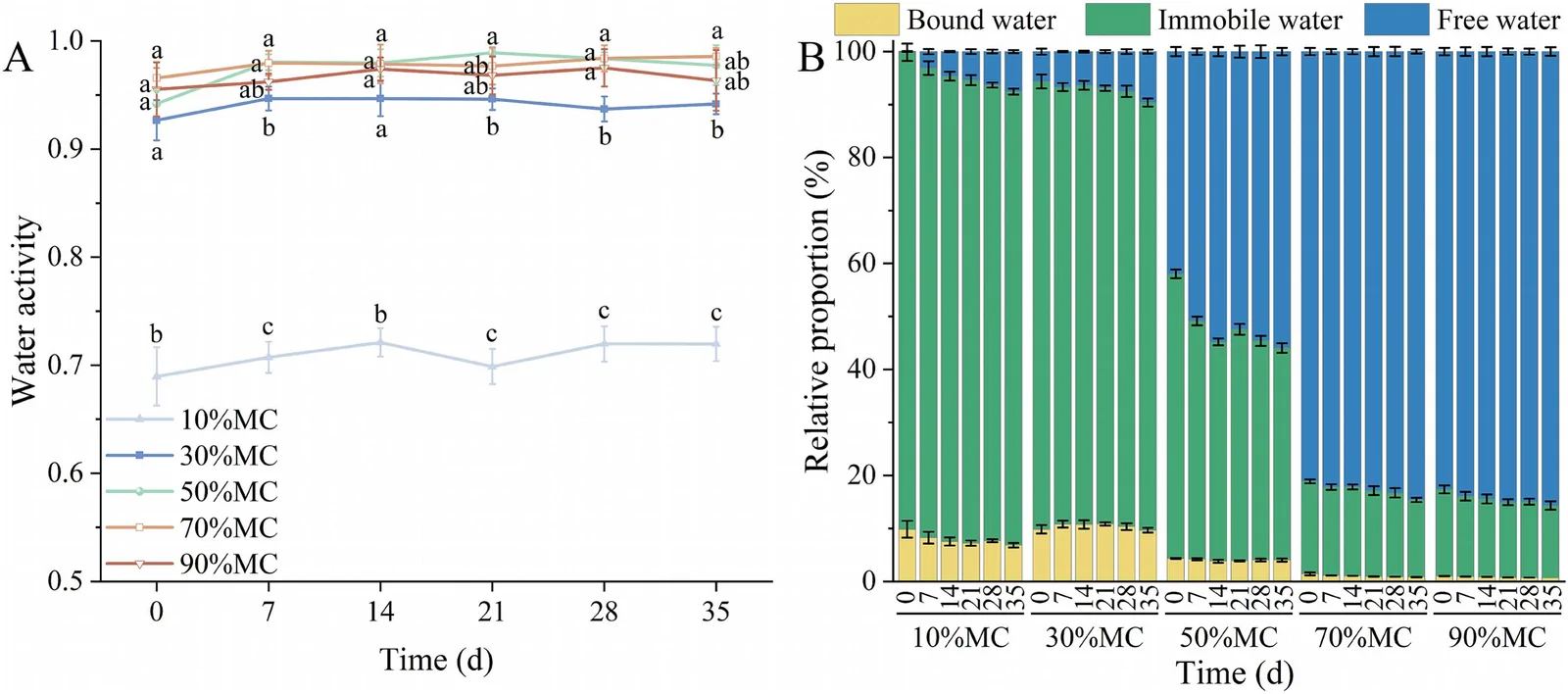
Effect of different moisture contents on the water activity (A) and water distribution (B) in the protein–lipid–water ternary systems during storage. Different letters indicate significant differences among groups (*p* < 0.05).

Water activity was positively correlated with moisture content; that is, higher moisture content corresponded to higher water activity. The water activity values of the 10% MC and 30% MC fluctuated slightly around 0.70 and 0.92, respectively. However, when the moisture content exceeded 50%, water activity showed only a slight and non-significant increase (*p* > 0.05), with all samples containing 50% MC or higher fluctuating around 0.96. This trend is consistent with the findings of Wang et al. (2020). Water activity is a key indicator of water mobility, and when the moisture content of a sample is sufficiently high, more water molecules exist as free water, and the system approaches moisture saturation, causing water activity to approach 1.0 and become relatively stable (Wang et al., 2025a).

#### 3.3.2 Water distribution

The changes in free water, immobile water, and bound water contents in different samples during oxidation were determined, as shown in Fig. 3 B, and the detailed data are provided in Table S3 in the Supplementary Materials.

The results showed that almost all samples exhibited an increase in free water content accompanied by decreases in immobile water and bound water contents. This phenomenon may be mainly attributed to the progressive disruption of protein structure as oxidation proceeded, which impaired the hydrated adsorption network and led to the conversion of a portion of immobile and bound water into free water (Xie et al., 2026). Notably, the immobile water content in the 50% MC decreased significantly from 53.71% to 40.00%, which is consistent with the water exudation phenomenon observed in Section 3.1. In contrast, the immobile water content of the 30% MC sample showed a slight increase during the early oxidation stage (0–14 d), possibly because mild oxidation during early storage induced partial unfolding of protein molecules and exposed more polar groups, thereby temporarily enhancing water adsorption capacity (Fang et al., 2022).

In addition, free water content was positively correlated with total moisture content, whereas the relative proportion of immobile water decreased with increasing total moisture content. The free water content in the 50% MC reached 40%, while those in the 70% MC and 90% MC samples exceeded 80%. This phenomenon is consistent with the findings of Chbani et al. (2020), who reported that once the binding sites on hydrophilic matrices such as proteins become saturated, additionally introduced water can no longer be strongly adsorbed and mainly exists as free water in the system.

Therefore, we speculate that, for samples with low to intermediate moisture contents (10–50% MC), differences in oxidation indicators may be mainly attributed to differences in water activity. By contrast, for samples with intermediate to high moisture contents (50–90% MC), although their water activities were similar, differences in oxidation indicators may primarily arise from variations in the content of free water. As discussed in Section 3.2, an appropriate amount of water can enhance molecular mobility, free radical diffusion, and interactions between proteins and lipid oxidation products, whereas excessive water may dilute reactants and restrict oxygen diffusion.

### 3.4 Protein structure

#### 3.4.1 Secondary structure of protein

As shown in Fig. 4 A and Table S4 in the supplementary materials, all samples exhibited a continuous decrease in α-helix content and varying degrees of increase in β-sheet and random coil contents with increasing oxidation time. During oxidation, the hydrogen-bonding network responsible for maintaining the α-helix structure is progressively disrupted, causing the ordered helical structure to unfold and transform into more extended β-sheet or random coil conformations. This transition is a characteristic feature of the conversion of protein secondary structure from a compact to a more disordered state (Fang et al., 2023). In addition, oxidative modification of protein side chains may increase surface hydrophobicity, thereby further promoting structural unfolding (Dominguez et al., 2022).

**Fig. 4:**
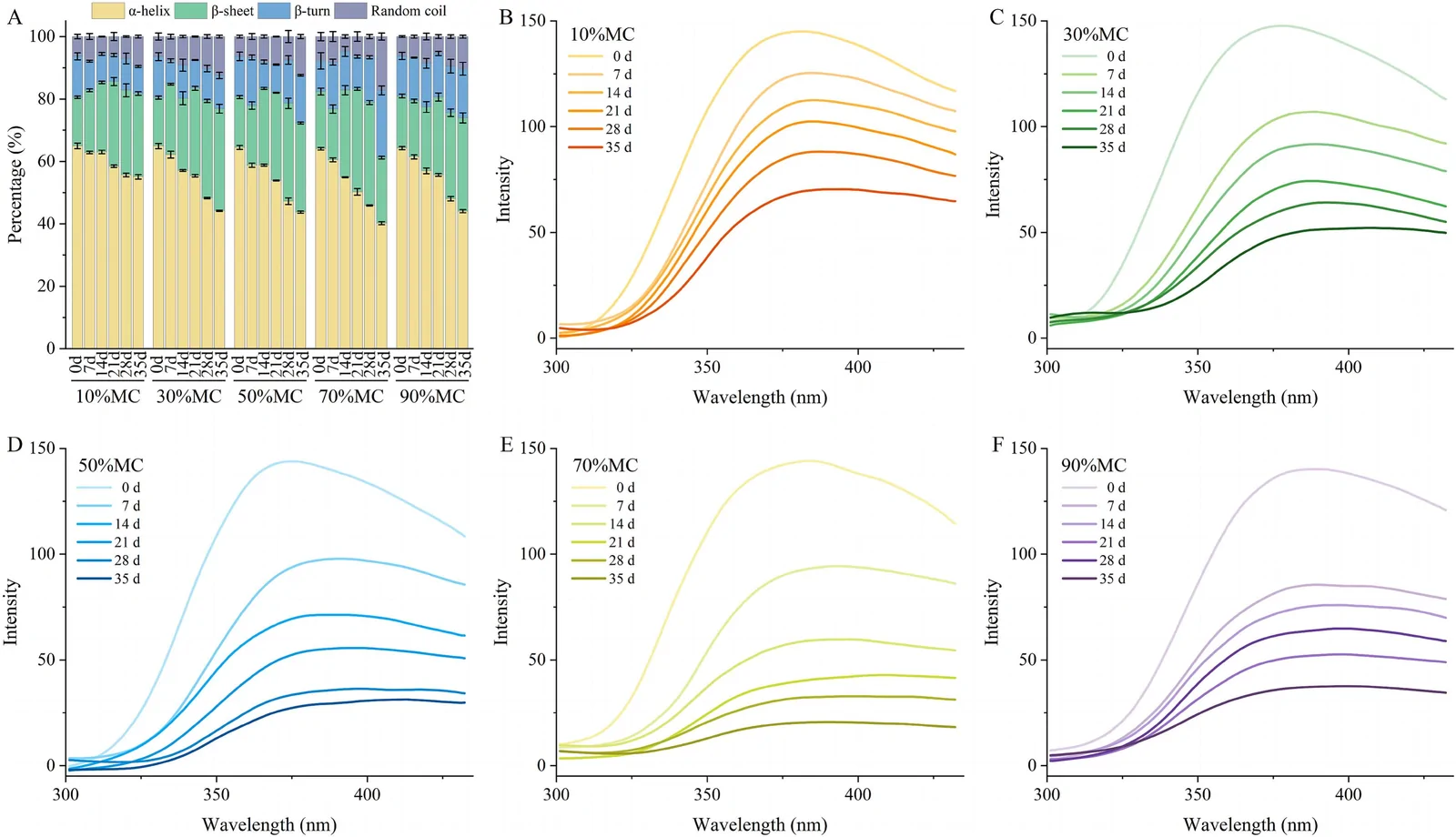
Effects of moisture content on the time-dependent changes in protein secondary and tertiary structures during storage. (A) Secondary structure; (B—F) tertiary structure at 10%, 30%, 50%, 70%, and 90% MC, respectively.

Overall, the extent of secondary structural changes increased with moisture content. Among all samples, the 70% MC exhibited the greatest decrease in α-helix content, declining continuously from 64.07% to 40.24%, accompanied by more pronounced increases in β-sheet and random coil contents. At 35 d, the marked decrease in β-sheet content together with the substantial increase in random coil content suggested extensive oxidation-induced structural disordering, aggregation, and precipitation (Dominguez et al., 2022). These structural alterations were consistent with the higher integrated oxidation level observed at 70% MC and were likely associated with its favorable free-water environment for molecular mobility and oxidative reactions.

The moisture-dependent structural evolution may result from the competition between hydration-induced plasticization and oxidation-related structural disruption. At low moisture content (10% MC), limited protein hydration and restricted molecular mobility may constrain conformational rearrangement and reduce the accessibility of oxidation-sensitive residues, thereby maintaining a relatively ordered secondary structure (Palacios et al., 2019). With increasing moisture content, water molecules can interact with polar groups in the protein matrix and weaken protein–protein hydrogen bonding and other non-covalent interactions, thereby acting as a plasticizer and increasing chain mobility and conformational flexibility (Zhang et al., 2022). Such hydration-induced relaxation may facilitate the exposure of previously buried oxidation-sensitive residues and increase their accessibility to reactive species, promoting the disruption and rearrangement of the hydrogen-bonding network and consequently accelerating the conversion of α-helices into β-sheet and random-coil structures (Pan et al., 2023). However, when moisture content increases further to 90%, the structural deterioration does not increase proportionally. Excess water may increase the spatial separation of reactants and dilute reactive species and pro-oxidants, thereby decreasing the probability of effective oxidative interactions (Ghelichi et al., 2023; Wang et al., 2025b). Therefore, the pronounced structural deterioration observed at 70% MC may reflect a balance in which sufficient molecular mobility and reactant accessibility coexist with a relatively high effective concentration of oxidative species.

#### 3.4.2 Tertiary structure of protein

Based on the sensitivity of tryptophan residues to changes in the polarity of their microenvironment, alterations in protein tertiary structure were investigated, and the results are shown in Fig. 4 B—F. At 0 d, all samples exhibited a maximum emission wavelength (λ_max_) of approximately 355 nm, which is characteristic of tryptophan fluorescence (Brack et al., 2024). As oxidation progressed, all samples displayed a red shift in λ_max_ to approximately 380 nm, accompanied by a gradual decrease in fluorescence intensity. These changes indicate that tryptophan residues became increasingly exposed to the aqueous phase during protein unfolding, resulting in an increase in microenvironmental polarity and a corresponding shift in λ_max_. In addition, the reduction in fluorescence intensity is generally attributed to water quenching effects caused by the exposure of tryptophan residues to a polar environment and may also reflect oxidative damage to tryptophan side chains (Chang et al., 2019).

Interestingly, the 90% MC exhibited the largest decrease in fluorescence intensity after 7 d of oxidation, suggesting that a high-moisture environment facilitated the unfolding of protein tertiary structure during the early stage of oxidation. This phenomenon may be associated with an enhanced protein hydration effect under high-moisture conditions, whereby water molecules penetrate into the protein interior, promote structural relaxation, and weaken the hydrophobic interactions and hydrogen-bonding network responsible for maintaining tertiary structural stability (Morón, 2021). However, as oxidation progressed, the rate of fluorescence decline in the 90% MC gradually slowed. By 35 d, its overall decrease in fluorescence intensity was lower than those observed in the 70% MC and 50% MC but remained higher than those of the 30% MC and 10% MC. This phenomenon may be related to the higher oxidation levels of the 70% MC and 50% MC during the later stages of storage, as discussed in Section 3.2. More intense oxidation can promote oxidative modification of protein surfaces, exposure of hydrophobic groups, and increased surface hydrophobicity, thereby accelerating the further unfolding of protein tertiary structure during the later stages of oxidation (Zhang et al., 2024).

Furthermore, the fluorescence results were generally consistent with the secondary-structure analysis. As moisture content increases, water molecules can interact with polar groups of proteins and alter hydrogen bonds, hydrophobic interactions, and other non-covalent forces that maintain the native protein structure (Morón, 2021). This may increase the mobility and flexibility of protein chains, facilitating structural relaxation and the exposure of buried tryptophan residues and other oxidation-sensitive groups (Zhang et al., 2024). The increased exposure of these residues may, in turn, make them more accessible to reactive species, thereby promoting oxidative modification and further protein unfolding (Pan et al., 2023). However, excessive water can increase the spatial separation between reactants and reduce the local concentrations of reactive species and pro-oxidants, thereby decreasing the probability of effective oxidative interactions (Ghelichi et al., 2023). Therefore, the effect of moisture on protein structure may depend on a balance between enhanced protein molecular mobility and effective oxidative activity.

### 3.5 Levels of free radicals

O₂•^−^ and •OH are important reactive oxygen species involved in oxidation processes. The former is mainly derived from the one-electron reduction of molecular oxygen and lipid radical chain reactions, whereas the latter is primarily generated through the conversion of superoxide anion radicals, decomposition of lipid hydroperoxides, and Fenton reactions involving H₂O₂ (Ruzza et al., 2021).

As shown in Fig. 5 A–B, the generation of both O₂•^−^ and •OH exhibited clear moisture-dependent patterns. The content of O₂•^−^ generally showed an initial increase followed by a subsequent decrease during oxidation. The 50% MC and 70% MC both reached peak values at 21 d, with the 70% MC exhibiting the highest peak value of approximately 2.37 μmol/mg, which was significantly higher than those of the other treatments. Thereafter, the content of O₂•^−^ gradually decreased, possibly due to its further conversion into hydroxyl radicals or participation in subsequent oxidation reactions (Deng et al., 2026). This indicates that O₂•^−^ is more likely to function as a reactive intermediate during the early and middle stages of oxidation rather than continuously accumulating in the system.

**Fig. 5:**
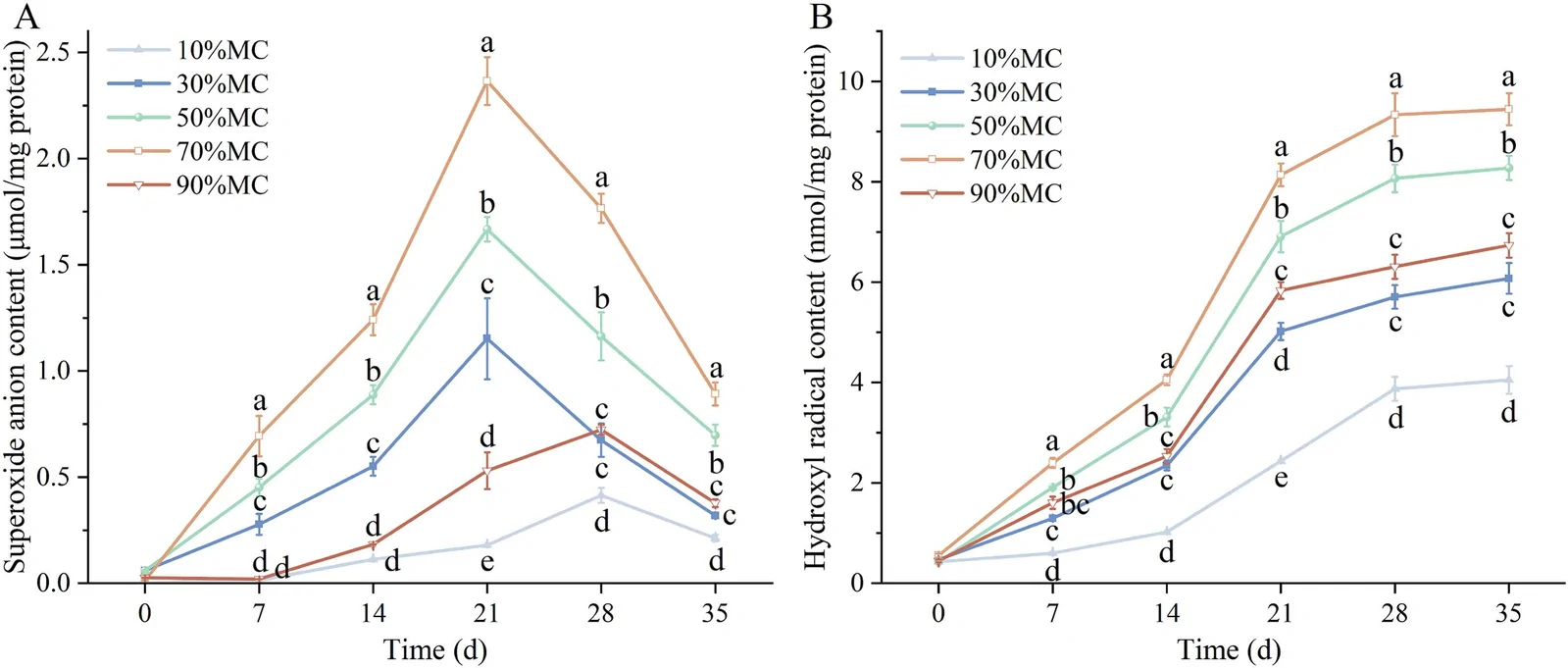
Effects of moisture content on reactive oxygen species levels during storage. (A) Superoxide anion radical (O₂•^−^); (B) hydroxyl radical (•OH). Different letters indicate significant differences among groups (*p* < 0.05).

In contrast, the content of •OH increased continuously throughout the oxidation process and reached its maximum at 35 d. At the later stage of oxidation, •OH levels among the different moisture groups generally followed the order 70% MC > 50% MC > 90% MC > 30% MC > 10% MC, indicating that an appropriate amount of water markedly promoted the continuous generation of hydroxyl radicals. This may be attributed to enhanced molecular mobility, promoted diffusion of oxygen and free radicals, and increased contact probability between proteins and lipid oxidation products (Zhang et al., 2025). Overall, the free-radical profiles were consistent with the integrated oxidation indices, indicating that intermediate moisture favored reactive oxygen species formation and propagation and thereby facilitated protein–lipid co-oxidation.

In addition, consistent with the previous speculation, the O₂•^−^ and •OH levels in the 90% MC remained at intermediate levels, indicating that excessive moisture inhibited free radical generation and accumulation to some extent. Notably, the content of O₂•^−^ in the 90% MC was only slightly higher than that in the 10% MC, whereas its •OH content was significantly higher than that in the 10% MC. This suggests that the formation and accumulation mechanisms of these two reactive oxygen species may differ under high-moisture conditions. We speculate that excessive moisture mainly inhibited the continuous accumulation of O₂•^−^ by diluting reactants and restricting oxygen diffusion into the lipid phase (Bülbül et al., 2020), whereas its inhibitory effect on the subsequent conversion of O₂•^−^ and Fenton-type reaction-mediated •OH formation was relatively weaker. Therefore, under the 90% MC condition, the O₂•^−^ level was close to that of the 10% MC, while the •OH level remained relatively high.

### 3.6 Multivariate statistical analysis

#### 3.6.1 Correlation analysis

Correlations among different indicators were further analyzed, as shown in Fig. 6 A. The results showed that moisture content was highly significantly correlated with both water activity and water composition (*p* < 0.001), indicating that different moisture contents markedly altered the water distribution characteristics of the system. Specifically, moisture content was significantly positively correlated with water activity and free water content, with correlation coefficients of 0.74 and 0.95, respectively, whereas it was significantly negatively correlated with bound water and immobile water contents, with correlation coefficients of −0.87 and − 0.96, respectively.

**Fig. 6:**
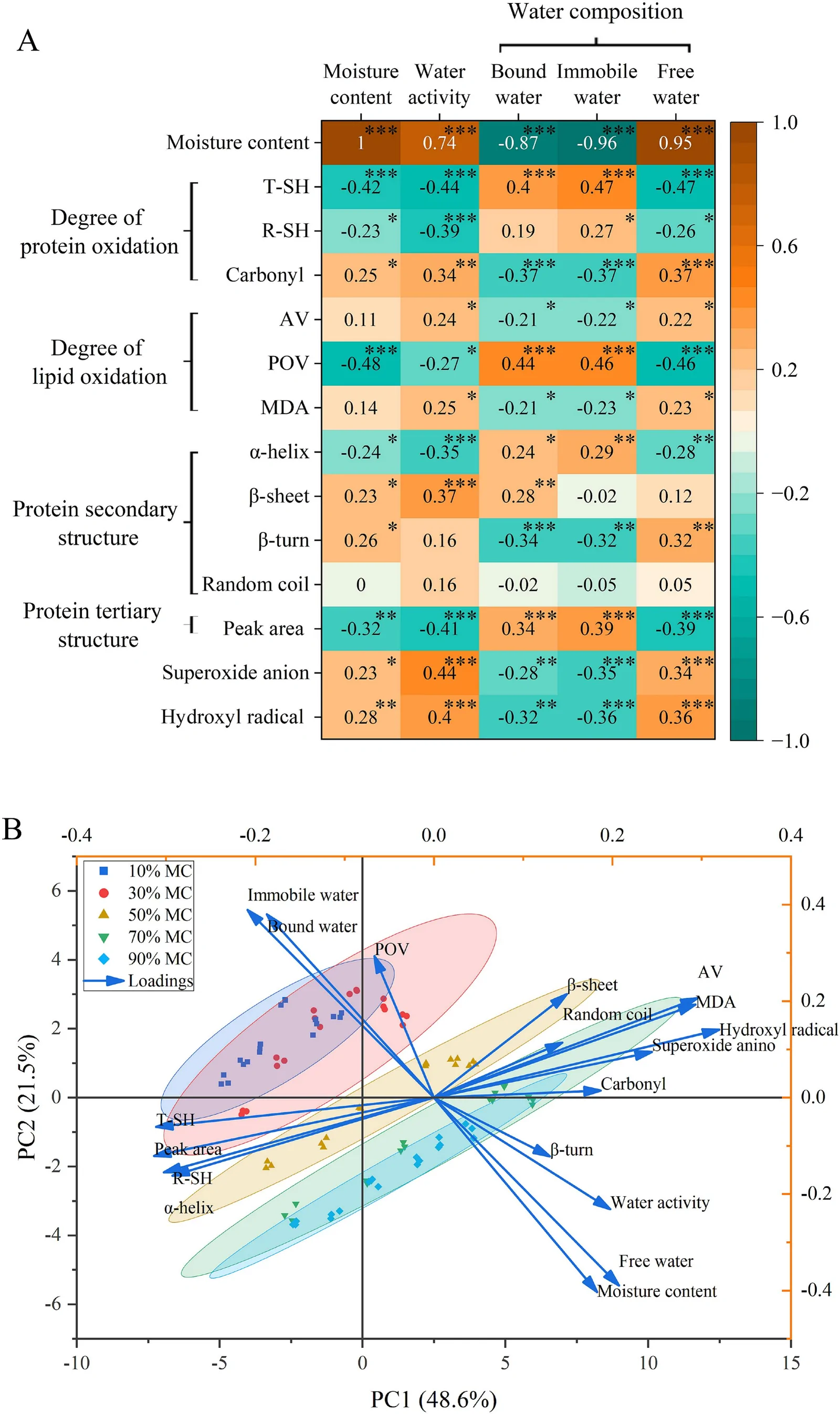
Multivariate analysis of moisture-dependent protein–lipid co-oxidation. (A) Correlation analysis among moisture-related parameters, protein and lipid oxidation indicators, protein structural characteristics, and free radical levels; (B) principal component analysis (PCA) of the measured variables. *, **, and *** represent *p* < 0.05, *p* < 0.01, and *p* < 0.001, respectively.

Further comparison of the correlations between moisture-related indicators and oxidation indicators, protein structural parameters, and free radical levels revealed that water activity and water composition differed in their effects on oxidation and structural changes. Overall, water activity showed stronger correlations with R-SH content, AV, MDA, α-helix, β-sheet, tertiary structure, O₂•^−^, and •OH levels than water distribution indicators, suggesting that water availability plays an important role in promoting molecular mobility, free radical diffusion, and protein conformational relaxation. In contrast, water distribution showed stronger correlations with T-SH content, carbonyl content, POV, and β-turn, indicating that water state may have a more direct influence on protein oxidative modification and the accumulation of primary lipid oxidation products.

Notably, compared with moisture content itself, moisture-related indicators such as water activity, bound water, immobile water, and free water generally exhibited more numerous and stronger significant correlations with oxidation and structural indicators, including carbonyl content, AV, MDA, T-SH, tertiary structure, O₂•^−^, and •OH levels. This suggests that moisture content is more likely to affect protein–lipid co-oxidation by regulating water activity and water distribution, thereby altering the migration and contact efficiency among oxygen, free radicals, lipid oxidation products, and protein molecules.

#### 3.6.2 Principal component analysis

To further elucidate the overall relationships between moisture content and protein–lipid co-oxidation, PCA was performed based on the measured oxidation-related indicators, and the results are presented in Fig. 6 B. The first two principal components accounted for 70.1% of the total variance, with PC1 and PC2 explaining 48.6% and 21.5%, respectively, indicating that the PCA model effectively distinguished the overall differences among systems with different moisture contents. The score plot showed a clear separation of the samples according to moisture content, which were distributed sequentially along the PC1 axis in the order of 10%, 30%, 50%, 70%, and 90% MC, suggesting that moisture content was the dominant factor governing protein–lipid co-oxidation.

The loading plot further revealed that the 10% and 30% MC samples were strongly associated with bound water, immobile water, α-helical, T-SH, and R-SH contents, while being located opposite to oxidation-related variables, including carbonyl content, MDA, AV, free radicals, and β-sheet. These results indicate that under low-moisture conditions, restricted molecular mobility together with a relatively stable protein conformation effectively inhibited lipid oxidation and the subsequent protein oxidation induced by lipid-derived reactive species. In contrast, the 90% MC sample was closely associated with free water, water activity, and moisture content, but showed relatively weak correlations with the oxidation-related indicators. This finding suggests that excessive moisture also suppressed protein–lipid co-oxidation, possibly because of reduced reactant concentrations and the dilution of reactive radicals.

Notably, the intermediate-moisture samples (50% and 70% MC) were clustered in the direction of the oxidation-related loadings and exhibited strong associations with MDA, AV, hydroxyl radicals, superoxide anion radicals, carbonyl content, and β-sheet structure. Among them, the 70% MC sample was located closest to these oxidation-related variables, indicating that intermediate moisture conditions were most favorable for the generation of lipid oxidation products and their subsequent transfer to proteins, thereby promoting protein–lipid co-oxidation. Collectively, these results also further demonstrate the dual regulatory role of moisture content in protein–lipid co-oxidation: both excessively low and excessively high moisture contents suppressed the co-oxidation process, whereas intermediate moisture represented the most active transition region for protein–lipid co-oxidation.

### 3.7 Validation in real sturgeon matrices

To evaluate whether the oxidation behavior observed in the ternary model could be extrapolated to a real food matrix, native sturgeon meat (NSM) and composition-matched sturgeon meat (CSM) were included as validation samples. Besides, the moisture contents of NSM and CSM were comparable to that of the 70% MC model system. As discussed previously, the 70% MC system exhibited the highest degree of protein–lipid co-oxidation among all model systems investigated. Therefore, changes in protein oxidation, lipid oxidation, protein secondary structure, and free radical levels during storage were systematically evaluated in NSM and CSM, and compared with those in the 70%MC model system, as shown in Fig. 7.

**Fig. 7:**
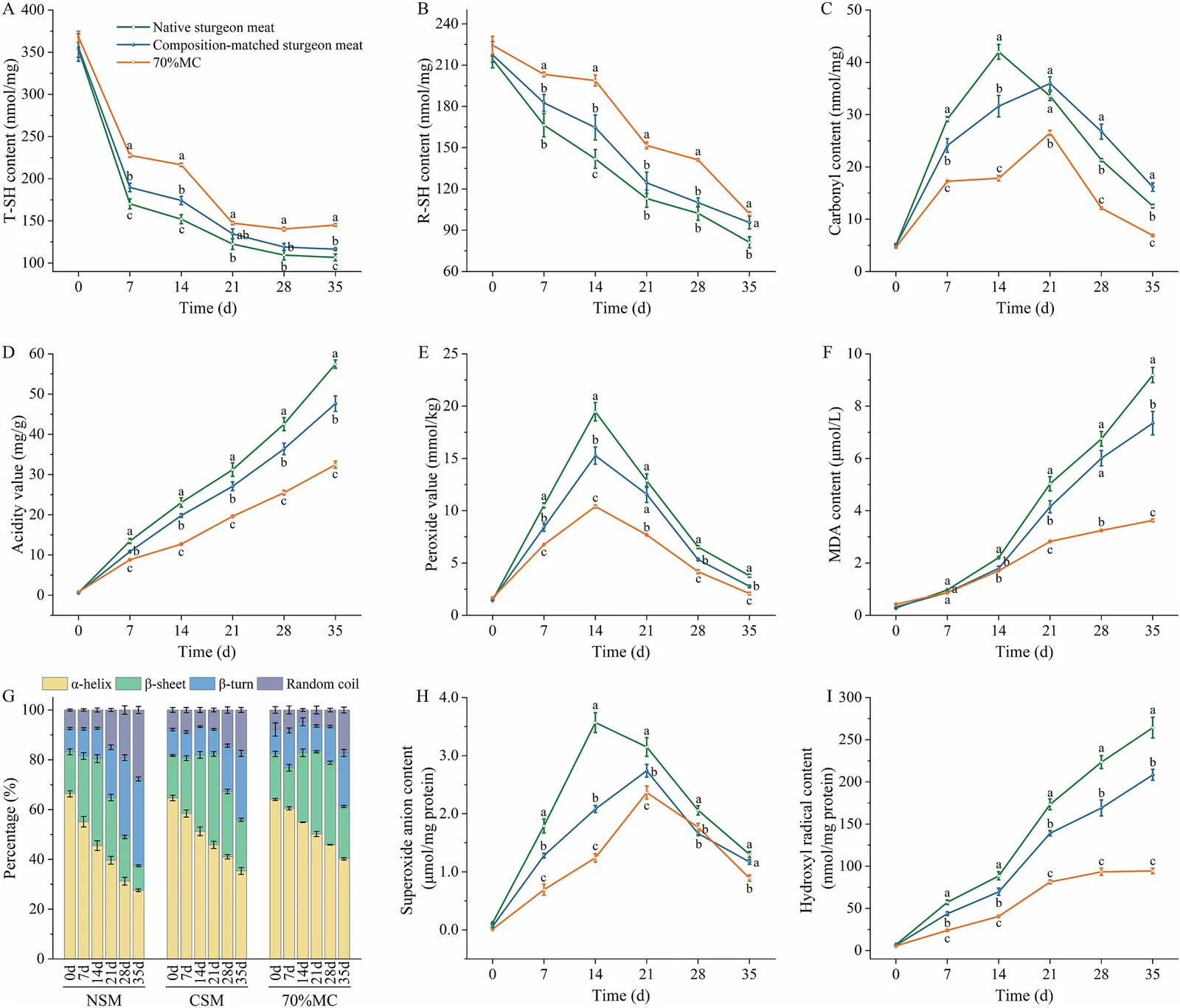
Validation of the ternary model using real sturgeon matrices during storage. Comparative changes in protein oxidation, lipid oxidation, protein secondary structure, and free radical levels in native sturgeon meat (NSM), composition-matched sturgeon meat (CSM), and the 70% MC model system. (A) T-SH; (B) R-SH; (C) carbonyl content; (D) acid value; (E) peroxide value; (F) MDA content; (G) protein secondary structure; (H) O₂•^−^; and (I) •OH. Different letters indicate significant differences among groups (*p* < 0.05).

Compared with the 70%MC model system, both NSM and CSM exhibited significantly greater protein and lipid oxidation (also shown in Table 1), more pronounced protein structural changes, and higher free radical levels during storage. This difference may be attributed to the presence of various endogenous factors in real sturgeon meat—including pro-oxidants such as heme proteins, non-heme iron, oxidative enzymes, and membrane phospholipids, as well as antioxidants like α-tocopherol and glutathione (Wu et al., 2024) —all of which are largely absent or substantially diminished in the simplified ternary model system. Notably, numerous studies have demonstrated that in post-mortem fish muscle, the pro-oxidative effect of these endogenous components far outweighs the protective capacity of the antioxidants, which are rapidly exhausted during the early stages of storage (Dominguez et al., 2022; Nawaz et al., 2022). Furthermore, NSM exhibited more severe oxidative deterioration than CSM, which may be attributed to the retention of greater amounts of endogenous pro-oxidative factors in NSM, as well as its higher lipid content, thereby providing a larger pool of oxidation-susceptible substrates.

Despite the quantitative differences in oxidation intensity, the temporal evolution of all measured indicators was highly consistent between the real samples and the 70% MC model system. Compared with the 70% MC, the real samples generally exhibited higher peak values, earlier peak occurrence, and faster increases or declines in the measured parameters, which can be attributed to their more extensive oxidative deterioration. Besides, the 70%MC model exhibited the highest POI/LOI ratio, followed by CSM and NSM (Table 1), which may be explained by the disproportionately stronger pro-oxidative effects of these endogenous factors on lipids than on proteins. Consistent with this interpretation, the increase in LOI in the real samples was substantially greater than the corresponding increase in POI, as shown in Table 1. These findings demonstrate that the ternary model successfully reproduces the overall progression of protein–lipid co-oxidation in real sturgeon muscle. Therefore, although the simplified model cannot fully capture the oxidation intensity of the complex food matrix, it provides a reliable platform for elucidating the dynamic behavior of protein–lipid co-oxidation.

## 4 Conclusion

This study established a sturgeon myofibrillar protein–sturgeon oil–water ternary model to elucidate the effect of moisture content on protein–lipid co-oxidation. Both protein and lipid oxidation exhibited a non-linear response to increasing moisture content, with oxidation first intensifying and then declining, and the most severe co-oxidation occurring at 70% MC. Low moisture restricted molecular mobility and reactant diffusion, whereas excessive moisture reduced effective reactant concentrations and may have limited oxygen transfer, thereby attenuating the overall co-oxidation process rather than completely suppressing oxidative reactions. In contrast, intermediate moisture provided sufficient free water to facilitate molecular movement, radical propagation, and the transfer of lipid-derived reactive products to proteins. The pronounced structural disruption and free-radical accumulation observed at 70% MC further confirmed that intermediate moisture created the most favorable conditions for protein–lipid co-oxidation.

Multivariate statistical analysis further demonstrated that intermediate-moisture conditions were most favorable for the generation of lipid oxidation products and their subsequent transfer to proteins, thereby promoting protein–lipid co-oxidation. Moisture content may regulate this process by modulating moisture-related parameters, including water activity and the proportions of free, immobilized, and bound water, which in turn influence protein conformational changes, free-radical generation, lipid oxidation, and protein oxidation. Collectively, these findings indicate that both excessively low and excessively high moisture contents resulted in lower overall protein–lipid co-oxidation than intermediate moisture, whereas intermediate moisture represented the most active moisture range for co-oxidation. Notably, the attenuation observed at high moisture did not imply complete inhibition of oxidative reactions, as certain reactive species, particularly ·OH, remained at appreciable levels. This moisture-dependent pattern suggests the possible existence of a transition mechanism and potentially a critical threshold, most likely within the 40–60% moisture range.

Finally, validation using real sturgeon matrices demonstrated that the ternary model established in this study successfully reproduced the overall progression of protein–lipid co-oxidation observed in real samples, although it underestimated the absolute oxidation intensity. The more extensive protein and lipid oxidation detected in the real samples was primarily attributed to the presence of endogenous pro-oxidative components. Nevertheless, the consistent temporal trends observed between the model and real samples support the applicability of this system for providing mechanistic insights and practical guidance for the development and oxidative stabilization of commercial marine protein products.

## CRediT authorship contribution statement

**Yizhou Fang:** Writing – original draft, Visualization, Validation, Methodology, Conceptualization. **Zhengmei Yin:** Methodology, Data curation. **Jinshui Yu:** Writing – original draft, Data curation. **Guangrong Huang:** Writing – review & editing, Writing – original draft. **Junhui Li:** Writing – review & editing, Validation, Supervision, Funding acquisition.

## Declaration of competing interest

The authors declare that they have no known competing financial interests or personal relationships that could have appeared to influence the work reported in this paper.

## Data Availability

Data will be made available on request.
