# Supplementary material for: Effects of moisture content on the co-oxidation behavior of sturgeon myofibrillar protein and sturgeon oil
Source: Food Chem X. 2026 Sep 16;39:104481. doi: 10.1016/j.fochx.2026.104481 (PMC13631886; doi:10.1016/j.fochx.2026.104481)
Supplement: Supplementary file 1 [file mmc1.docx]

Figure s1. Effect of different moisture contents (10%-90% MC) on the observed rates of change (Increase/Decay) of myofibrillar protein oxidation (A: Total sulfhydryl content; B: Reactive sulfhydryl content; C: Carbonyl content) and lipid oxidation (D: Acid value; E: Peroxide value; F: Malondialdehyde content) over time

Figure s2. Changes in moisture content (%) of samples with different initial moisture contents during storage

Table s1. General composition of the prepared samples with different moisture contents

| Composition | Protein-lipid-water systems* | | | | | Native sturgeon meat | Composition-matched sturgeon meat |
| --- | --- | --- | --- | --- | --- | --- | --- |
|  | 10%MC | 30%MC | 50%MC | 70%MC | 90%MC |  |  |
| Moisture (%) | 10.29±0.44^a^ | 29.98±0.36^b^ | 50.56±1.12^c^ | 69.13±0.90^d^ | 90.33±1.28^e^ | 72.44±1.37^f^ | 70.83±1.02^df^ |
| Protein (%) | 80.52±1.35^a^ | 63.18±1.30^b^ | 44.98±1.19^c^ | 27.17±1.10^d^ | 8.91±0.67^e^ | 18.89±0.62^f^ | 25.81±0.81^d^ |
| Lipids (%) | 7.97±0.08^a^ | 6.41±0.12^b^ | 4.63±0.17^c^ | 2.72±0.04^d^ | 0.92±0.03^e^ | 4.61±0.10^c^ | 2.57±0.07^f^ |
| Protein : Lipid | 9.97±0.21^a^ | 9.86±0.13^a^ | 9.72±0.11^a^ | 9.99±0.25^a^ | 9.72±0.47^a^ | 4.10±0.05^b^ | 10.03±0.25^a^ |

* MC: moisture content;

Different letters indicate significant differences among groups (*p* < 0.05).

Table s2. Effects of 10 kGy γ-irradiation on the initial oxidative status and structural characteristics of sturgeon myofibrillar protein and sturgeon oil

| Parameter | 10 kGy γ-irradiation | |
| --- | --- | --- |
|  | Before | After |
| **Protein oxidation** |  |  |
| Carbonyl content (nmol/mg) | 4.49±0.20^a^ | 4.87±0.45^a^ |
| T-SH content (nmol/mg) | 370.25±4.69^a^ | 354.51±5.21^b^ |
| R-SH content (nmol/mg) | 258.87±6.36^a^ | 243.70±9.23^a^ |
| **Protein secondary structure** |  |  |
| α-Helix (%) | 66.16±0.47^a^ | 64.84±0.63^b^ |
| β-Sheet (%) | 15.05±0.43^a^ | 15.61±0.61^a^ |
| β-Turn (%) | 13.51±0.30^a^ | 13.24±0.14^a^ |
| Random coil (%) | 5.28±0.57^a^ | 6.30±0.47^a^ |
| **Lipid oxidation** |  |  |
| Acidity value (mg/g) | 0.77±0.03^a^ | 0.81±0.03^a^ |
| Peroxide value (mmol/kg) | 1.61±0.13^a^ | 1.71±0.08^a^ |
| MDA content (μmol/L) | 0.39±0.02^a^ | 0.43±0.02^a^ |
| **Total viable count (CFU/g)** |  |  |
| In protein | 6744±180^a^ | <10^b, *^ |
| In oil | 609±28^a^ | <10^b^ |

Different letters indicate significant differences among groups (*p* < 0.05);

* A result of <10 CFU/g indicates that no colonies were observed, falling below the detection limit of the plate count method according to 990.12 of AOAC (2023).

Table s3. Changes in the relative content of three water states in protein–lipid–water ternary systems with different moisture contents during storage

| Samples^*^ | Water states | Time (d) | | | | | |
| --- | --- | --- | --- | --- | --- | --- | --- |
|  |  | 0 | 7 | 14 | 21 | 28 | 35 |
| 10%MC | Bound Water | 9.83±1.58^Aa^ | 8.26±1.13^Aa^ | 7.53±0.76^Aa^ | 7.23±0.50^Aa^ | 7.69±0.27^Aa^ | 6.84±0.44^Aa^ |
|  | Immobile Water | 90.04±1.65^Aa^ | 88.63±1.30^Aab^ | 87.84±0.84^Abc^ | 87.38±0.95^Abcd^ | 86.00±0.47^Acd^ | 85.60±0.56^Ae^ |
|  | Free Water | 0.14±0.06^Aa^ | 3.11±0.49^Ab^ | 4.62±0.14^Ac^ | 5.39±0.46^Ad^ | 6.31±0.34^Ae^ | 7.56±0.30^Af^ |
| 30%MC | Bound Water | 9.84±0.81^Aa^ | 10.81±0.65^Ba^ | 10.76±0.79^Ba^ | 10.85±0.31^Ba^ | 10.34±0.64^Ba^ | 9.66±0.47^Ba^ |
|  | Immobile Water | 84.52±1.34^Ba^ | 82.50±0.76^Bb^ | 82.87±0.85^Bb^ | 82.24±0.50^Bbc^ | 82.16±1.06^Bbc^ | 80.73±0.77^Bd^ |
|  | Free Water | 5.63±0.58^Ba^ | 6.69±0.12^Bb^ | 6.37±0.16^Bb^ | 6.91±0.27^Bbc^ | 7.49±0.45^Ac^ | 9.61±0.35^Bd^ |
| 50%MC | Bound Water | 4.34±0.11^Ba^ | 4.19±0.21^Cab^ | 3.84±0.29^Cab^ | 3.91±0.13^Cab^ | 4.04±0.27^Cab^ | 4.03±0.30^Cb^ |
|  | Immobile Water | 53.71±0.84^Ca^ | 44.95±0.83^Cb^ | 41.37±0.63^Cc^ | 43.67±1.02^Cb^ | 41.38±0.94^Cc^ | 40.00±0.90^Cc^ |
|  | Free Water | 41.95±0.88^Ca^ | 50.86±0.63^Cb^ | 54.78±0.85^Cc^ | 52.42±1.10^Cb^ | 54.58±1.21^Bc^ | 55.97±0.69^Cc^ |
| 70%MC | Bound Water | 1.43±0.28^Ca^ | 1.12±0.03^Db^ | 1.05±0.02^Dbc^ | 0.93±0.03^Dac^ | 0.89±0.02^Dac^ | 0.81±0.03^Dac^ |
|  | Immobile Water | 17.45±0.39^Da^ | 16.70±0.53^Dab^ | 16.78±0.48^Dab^ | 16.21±0.84^Db^ | 15.84±0.88^Db^ | 14.57±0.40^Dc^ |
|  | Free Water | 81.12±0.67^Da^ | 82.18±0.49^Dab^ | 82.17±0.49^Dab^ | 82.86±0.87^Db^ | 83.27±0.89^Cb^ | 84.63±0.40^Dc^ |
| 90%MC | Bound Water | 1.02±0.02^Ca^ | 0.90±0.02^Eb^ | 0.86±0.01^Eb^ | 0.76±0.01^Ec^ | 0.72±0.01^Ec^ | 0.67±0.00^Dd^ |
|  | Immobile Water | 16.36±0.74^Da^ | 15.18±0.83^Dab^ | 14.70±0.86^Ebc^ | 14.18±0.57^Ebc^ | 14.34±0.58^Ebc^ | 13.65±0.80^Dc^ |
|  | Free Water | 82.62±0.72^Ea^ | 83.92±0.82^Eab^ | 84.44±0.85^Ebc^ | 85.07±0.58^Ebc^ | 84.94±0.57^Dbc^ | 85.68±0.81^Ec^ |

* MC: moisture content;

Uppercase letters (A, B, C) indicate significant differences among different groups at the same time point (p < 0.05), while lowercase letters (a, b, c) indicate significant differences among different time points within the same group (*p* < 0.05).

Table s4. Changes in the relative content of four types of protein secondary structures in protein–lipid–water ternary systems with different moisture contents during storage

| Samples^*^ | Water states | Time (d) | | | | | |
| --- | --- | --- | --- | --- | --- | --- | --- |
|  |  | 0 | 7 | 14 | 21 | 28 | 35 |
| 10%MC | α-helix | 64.97±0.87^Aa^ | 62.86±0.41^Ab^ | 63.02±0.58^Aab^ | 58.45±0.40^Ac^ | 55.67±0.59^Ad^ | 55.05±0.76^Ae^ |
|  | β-sheet | 15.59±0.35^Aa^ | 19.91±0.47^Ab^ | 22.29±0.40^Ac^ | 27.16±1.28^Ad^ | 27.13±2.09^Ad^ | 26.69±0.56^Ae^ |
|  | β-turn | 13.10±1.13^Aa^ | 9.36±0.33^Ab^ | 9.13±0.44^Ab^ | 8.48±0.53^Ab^ | 10.15±1.73^Ab^ | 8.69±0.32^Ab^ |
|  | Random coil | 6.34±0.74^Aa^ | 7.87±0.95^Ab^ | 5.56±0.11^Ac^ | 5.91±1.33^Ac^ | 7.05±0.26^Aab^ | 9.58±0.46^Ad^ |
| 30%MC | α-helix | 64.93±0.84^Aa^ | 62.15±1.08^Aa^ | 57.09±0.29^Bb^ | 55.41±0.44^Bc^ | 48.28±0.22^Bd^ | 44.23±0.20^Be^ |
|  | β-sheet | 15.51±0.48^Aa^ | 22.62±0.29^Bb^ | 23.14±2.06^Ab^ | 28.01±0.68^Ac^ | 31.09±0.53^Bd^ | 32.65±1.38^Bd^ |
|  | β-turn | 13.14±1.27^Aa^ | 7.50±0.61^Bb^ | 10.63±1.94^Bc^ | 9.10±0.13^Ac^ | 10.39±1.03^Ac^ | 10.67±1.04^Bc^ |
|  | Random coil | 6.42±1.12^Aa^ | 7.74±0.77^Aa^ | 9.14±0.40^Bb^ | 7.49±0.16^Ba^ | 10.24±0.62^Bb^ | 12.46±0.44^Bc^ |
| 50%MC | α-helix | 64.49±0.63^Aa^ | 58.79±0.66^Bb^ | 58.80±0.36^Bb^ | 53.90±0.14^Cc^ | 47.25±1.10^Bd^ | 43.76±0.36^Be^ |
|  | β-sheet | 16.16±0.49^Aa^ | 19.11±1.16^Ab^ | 24.59±0.36^Ac^ | 28.1±0.15^Ad^ | 31.38±1.65^Be^ | 28.55±0.32^Cd^ |
|  | β-turn | 13.02±1.36^Aa^ | 15.40±0.77^Cb^ | 8.46±0.64^Ab^ | 9.01±0.21^Ab^ | 13.89±1.37^Bab^ | 15.29±0.27^Cb^ |
|  | Random coil | 6.34±0.45^Aa^ | 6.69±0.97^Aa^ | 8.15±0.50^Bb^ | 8.99±0.11^Cb^ | 7.48±1.92^Aab^ | 12.40±0.70^Bc^ |
| 70%MC | α-helix | 64.07±0.41^Aa^ | 60.5±0.64A^Bb^ | 54.91±0.14^Cc^ | 50.22±1.05^Dd^ | 45.88±0.16^Ce^ | 40.24±0.48^Cf^ |
|  | β-sheet | 18.29±1.06^Ba^ | 16.27±1.34^Cb^ | 27.91±1.51^Bc^ | 33.02±0.46^Bd^ | 32.96±0.66^Bd^ | 20.99±0.43^De^ |
|  | β-turn | 9.84±2.64^Ba^ | 15.02±0.97^Cb^ | 12.43±1.59^Bc^ | 10.37±0.49^Aac^ | 14.53±0.47^Bbc^ | 21.52±1.41^Dd^ |
|  | Random coil | 7.80±1.33^Aa^ | 8.22±1.61^Ba^ | 4.75±0.56^Ab^ | 6.39±1.54^Aab^ | 6.63±0.70^Aab^ | 17.25±1.14^Cc^ |
| 90%MC | α-helix | 64.27±0.49^Aa^ | 61.47±0.66^Ab^ | 56.96±0.91^BCc^ | 55.65±0.46^Bc^ | 48.05±0.73^Bd^ | 44.04±0.51^Be^ |
|  | β-sheet | 16.69±0.61^Aa^ | 17.91±0.63^Ca^ | 20.45±1.71^Cb^ | 24.95±1.31^Cc^ | 27.55±1.18^Cd^ | 29.92±1.60^Cd^ |
|  | β-turn | 12.83±1.14^Aa^ | 13.89±0.12^Dab^ | 14.04±1.55^Cab^ | 13.97±0.66^Bab^ | 14.93±2.21^Bb^ | 15.72±2.05^Cb^ |
|  | Random coil | 6.21±0.30^Aa^ | 6.74±0.48^Aa^ | 8.55±1.09^Bb^ | 5.42±0.61^Ac^ | 9.46±0.61^Bbd^ | 10.32±0.49^Dd^ |

* MC: moisture content;

Uppercase letters indicate significant differences among different groups at the same time point (p < 0.05), while lowercase letters indicate significant differences among different time points within the same group (*p* < 0.05).
